# The Effect of Prophylactic Antipyretic Administration on Post-Vaccination Adverse Reactions and Antibody Response in Children: A Systematic Review

**DOI:** 10.1371/journal.pone.0106629

**Published:** 2014-09-02

**Authors:** Rashmi Ranjan Das, Inusha Panigrahi, Sushree Samiksha Naik

**Affiliations:** 1 Department of Pediatrics, All India Institute of Medical Sciences, Bhubaneswar, India; 2 Department of Pediatrics, Post-Graduate Institute of Medical Education and Research, Chandigarh, India; 3 Department of Obstetrics and Gynecology, SCB Medical College and Hospital, Cuttack, India; McGill University, Canada

## Abstract

**Background:**

Prophylactic antipyretic administration decreases the post-vaccination adverse reactions. Recent study finds that they may also decrease the antibody responses to several vaccine antigens. This systematic review aimed to assess the evidence for a relationship between prophylactic antipyretic administration, post-vaccination adverse events, and antibody response in children.

**Methods:**

A systematic search of major databases including MEDLINE and EMBASE was carried out till March 2014. Randomized controlled trials (RCTs) comparing prophylactic antipyretic treatment versus placebo post-vaccination in children ≤6 years of age were included. Two reviewers independently applied eligibility criteria, assessed the studies for methodological quality, and extracted data [PROSPERO registration: CRD42014009717].

**Results:**

Of 2579 citations retrieved, a total of 13 RCTs including 5077 children were included in the review. Prophylactic antipyretic administration significantly reduced the febrile reactions (≥38.0°C) after primary and booster vaccinations. Though there were statistically significant differences in the antibody responses between the two groups, the prophylactic PCM group had what would be considered protective levels of antibodies to all of the antigens given after the primary and booster vaccinations. No significant difference in the nasopharyngeal carriage rates (short-term and long-term) of *H. influenzae* or *S. pneumoniae* serotypes was found between the prophylactic and no prophylactic PCM group. There was a significant reduction in the local and systemic symptoms after primary, but not booster vaccinations.

**Conclusions:**

Though prophylactic antipyretic administration leads to relief of the local and systemic symptoms after primary vaccinations, there is a reduction in antibody responses to some vaccine antigens without any effect on the nasopharyngeal carriage rates of *S. pneumoniae* & *H. influenza* serotypes. Future trials and surveillance programs should also aim at assessing the effectiveness of programs where prophylactic administration of PCM is given. The timing of administration of antipyretics should be discussed with the parents after explaining the benefits & risks.

## Introduction

Though routine vaccination is extremely beneficial for children, one of the reasons for non-compliance of children is the adverse effect of the previous vaccination [1], [2]. Various side effects in the form of local (skin indurations, swelling, rash, pain, or erythema at injection site) and systemic reactions (fever, joint or muscle pain, vomiting, diarrhea, fainting, seizures, or other central nervous system effects) occur commonly after diphtheria, tetanus toxoids and pertussis (DTP) vaccination [3], [4]. Again, these reactions are more common after vaccination with whole cell pertusis component vaccine (DTwP) than with acellular pertusis component vaccine (DTaP). When the reactions occur, they usually occur within 24–48 hours following vaccination, are usually mild and self limited, but can result in discomfort in the child [3], [4]. It is a common practice for many health providers to suggest that an antipyretic be given preventively at the time of vaccine administration [5].

If the reactogenicity of these vaccines are decreased in the general population, parental anxiety could be relieved to some extent. But there have been different schools of thought regarding prophylactic antipyretic administration. A systematic review conducted way back in 2007 concluded that parents be counseled to monitor vaccine-related adverse reactions and to treat them if and when they occur [6]. This review summarized the findings pertaining only to DTP vaccination, and not to other childhood vaccinations. Recent clinical trials have found that although febrile reactions were significantly decreased by prophylactic antipyretics, antibody responses to several vaccine antigens were reduced [7], [8]. Meanwhile, the American Academy of Pediatrics (AAP) continues to say that either prophylactic or therapeutic use of antipyretics should not be withheld [9]. Therefore, the current systematic review was planned to bridge this gap of information and provide any recommendation on the use of prophylactic antipyretics post-vaccination in children based on the available evidence.

## Methods

The protocol was registered with PROSPERO (Registration number: CRD42014009717).

### Types of studies

Randomized controlled trials (RCTs)

### Types of participants

Children of both sex and ≤6 year age undergoing routine immunization were included. Children suffering from chronic debilitating diseases, severe malnutrition (weight for height <3SD), and underlying immunodeficiency were excluded because of unpredictability of the antibody response after immunizations.

### Types of interventions

The intervention commenced either before, or after the child had received any of the routine childhood vaccinations, and consisted of prophylactic or preventive administration of antipyretics (either paracetamol or ibuprofen or both) or placebo/no prophylactic antipyretics. All formulation, dose and schedule of administration of antipyretics were considered.

### Types of outcome measures

#### Primary outcome measures

(1) Febrile reactions ≥38.0°C (100.4°F) in the first 24–48 hrs of primary and booster vaccinations

(2) Antibody response rate [measured by geometric mean concentration (GMC)] after primary (2, 3, and 4 or 3, 4, and 5 months) and booster vaccinations (12–15 months, and 40–48 months)

#### Secondary outcome measures

(1) High febrile reactions ≥39.0°C in the first 24–48 hrs of primary and booster vaccinations

(2) Local symptoms (pain, redness, and swelling at the injection site) after primary and booster vaccinations

(3) Systemic symptoms (temperature, irritability/fussiness, drowsiness, diarrhea, vomiting, and loss of appetite) after primary and booster vaccinations

(4) Nasopharyngeal carriage (NPC) rate of the organisms (*S. pneumoniae*, *H. influenzae*, and others)

The same temperature cutoff was used to define the severity of fever in almost all the trials. All routes of temperature (oral, rectal, and axillary) measurements were considered.

Pain was graded as none, mild (light reaction to touch), moderate (protesting in response to touch or pain with limb movement), or severe (child resists limb movement or keeps limb immobile).

Seroprotection: defined as an antibody concentration ≥0.1 IU/mL for diphtheria and tetanus, 0.15 µg/mL for *H. influenzae* type b, and 10 mIU/mL for Hepatitis B.

Seropositivity: defined as, 5 ELISA U/mL for antibodies to acellular pertussis antigens; anti-pneumococcal serotypes 1, 4, 5, 6B, 7F, 9V, 14, 18C, 19F and 23F antibody concentrations ≥0.2 µg/mL (for PCV10); anti-polio type 1, type 2 and type 3 titres ≥8.

Booster vaccine response to PT, FHA and PRN, one month after the administration of the booster dose of DTPa combined vaccine was defined as appearance of antibodies in subjects who are seronegative (that is, with concentrations <5 ELU/mL) just before booster dose and at least two-fold increase of prevaccination antibody concentrations in those who are seropositive (that is, with concentrations ≥5 ELU/mL) just before booster dose.

For comparison purpose, an acceptable decreased immunogenicity of all the mentioned vaccines is that the final antibody concentrations should not be below the above mentioned seroprotective/seropositive titers after primary or booster vaccination series.

### Search methods for identification of studies

We searched the Cochrane Central Register of Controlled Trials (CENTRAL, The Cochrane Library, Issue 3, March 2014), which contains the Cochrane Acute Respiratory Infection (ARI) Group and the Cochrane Infectious Diseases Group Specialized Registers, Medline/Ovid (1970 – March 2014), Pubmed (1970 – March 2014), and Embase (1988 – December 2013).

For these database searching, a combination of following search terms were adopted: acetaminophen, paracetamol, ibuprofen, analgesics, antipyretics, adverse reactions, vaccination, immunization, DTwP, diphtheria tetanus–toxoid, whole-cell pertussis, DTaP, acellular pertussis, *Streptococcus pneumoniae*, *Haemophilus influenzae* type B, inactivated poliovirus, IPV, pneumococcal 7-valent conjugate, pneumococcal 10-valent conjugate, pneumococcal 13-valent conjugate, PCV, measles, mumps, rubella, MMR, meningococcal conjugate, varicella zoster, hepatitis A, hepatitis B, rotavirus, influenza, or pneumococcal polysaccharide. To identify RCTs, which results had remained unpublished; we searched the NIH clinical trial register (www.clinicaltrials.gov). Trials that focused on the therapeutic effects of antipyretics post-vaccination were excluded from the analysis. Articles obtained from this search were cross-referenced and bibliographies were checked for all relevant information. No language restrictions were applied. The search details are given in Appendix S1.

### Data extraction

Data extraction was done using a data extraction form that was designed and pilot tested *a priori*. Two authors independently extracted data from the included studies, including year, setting (country, setting, type of participants, vaccination schedule followed, type of vaccines administered), exposure/intervention (type of antipyretic, dose and schedule of administration, protocol deviation, type of placebo), results (outcome measures, effect, significance), and sources of funding/support. Disagreements in extracted data were resolved through discussion.

### Assessment of risk of bias in included studies

Two review authors independently assessed the methodological quality of the selected trials by using methodological quality assessment forms. We undertook quality assessment of the trials using the criteria outlined in the *Cochrane Handbook for Systematic Reviews of Interventions* [10]. Any disagreements between the two review authors were resolved by discussion with the third author. Trials were assessed with respect to the extent to which investigators minimised the potential for bias to occur and addressed other issues in relation to methodological quality. Publication bias that might affect the cumulative evidence was also assessed.

### Study descriptions

Information in relation to methodological quality, characteristics of participants, interventions and outcome measures of each trial is provided in **Table 1** [7], [8], [11]–[21].

**Table 1:** Characteristics of included studies.

Study, setting	Participants, vaccination	Intervention	Outcomes measured	Significant Finding
Ipp 1987; Canada (11)	DTwP (both primary and booster). N = 452. Age = 2–6 m, 18 m	Acetaminophen (P) 15 mg/kg/dose or placebo (C) given 0–30 min before vaccine, then 2 doses at 4 hr intervals.	Fever (>38.0°C), high fever (>39.0°C), redness, swelling, pain, drowsiness, fussiness, vomiting, anorexia, persistent crying unrelieved by cuddling), unusual crying (abnormal pitch).	Fever and high fever at 2–6 m; P vs C: 26.6% vs 43.5% and 3.3% vs 12.7% (p<0.0005 for both). Redness at 2–6 m; P vs C: 11.6% vs 20.4% (p<0.025). Pain (moderate to severe) at 2–6 m; P vs C: 16.3% vs 31.5% (p<0.001). Fussiness at 2–6 m; P vs C: 34.8% vs 58.8% (p<0.0001). Crying at 2–6 m; P vs C: 18.4% vs 30.1% (p<0.005). Anorexia at 2–6 m; P vs C: 6.9% vs 13.9% (p<0.05).
Lewis 1988; USA (12)	DTwP (both primary and booster). N = 282. Age = 2–6 m, 18 m, 4–6 y	Acetaminophen (P) 10 mg/kg or Placebo (C) given with vaccine, then 3, 7, 12, and 18 hrs after vaccination.	Fever (≥38°C), redness, swelling, induration, pain, drowsiness, anorexia, fussiness, vomiting, and crying (≥30 min).	Fever at 2–6 m and overall, P vs C: 30% vs 53% and 32% vs 53% (p<0.01 for both). Fussiness at 2–6 m and overall, with P vs C: 46% vs 72% and 48% vs 70% (p<0.01 for both).
Uhari 1988; Finland (13)	DTwP (primary). N = 263. Age = 5 m	Acetaminophen 75 mg or Placebo 1 dose 4 hr after vaccination	Fever (>37.5°C), fussiness, local reactions (not specified), drowsiness, diarrhea, and vomiting	None.
Diez-Domingo 1998; Spain (14)	DTwP (primary). N = 256. Age = 3 m, 5 m, 7 m	Ibuprofen prophylactically (P) 20 mg/kg/day given in 3 equal doses at 8 hr intervals or therapeutically (C) 7.5 mg/kg/dose when needed for adverse reactions.	Fever (≥38.0°C), pain, crying (persistent or unusual), drowsiness, fussiness, vomiting, diarrhea, anorexia, redness, edema, induration.	Temperature increase with age: 37.7±0.55, 37.9±0.68, and 38.0±0.92°C after 1st, 2nd, 3rd doses (p = 0.001). Induration, P vs C: 35.7% vs 44.4% (p<0.05). Pain, P vs C: 37.5% vs 41.9% (p<0.05). Crying, P vs C: 16. 3% vs 27.5% (p<0.05). Drowsiness, P vs C: 30.1% vs 36.9% (p = 0.051). Fussiness, P vs C: 25.4% vs 37.7% (p<0.05).
Jackson 2006; USA (15)	DTaP (booster). N = 372. Age = 4–6 yrs	Acetaminophen 15 mg/kg up to 450 mg, Ibuprofen 10 mg/kg up to 300 mg, or Placebo given at vaccination; 2 doses following at 6 hr intervals.	Primary outcomes: local reactions (area of redness or discoloration in the vaccinated limb during the 2 days after vaccination, increase in mid-limb circumference during the 2 days after vaccination), and a persistent reaction (area of redness or discoloration present on the third day after vaccination). Secondary outcomes: Fever ≥38.0°C (during the next 2 days), local reactions (area of redness or discoloration in the vaccinated limb during the next 6 days after vaccination), itching (during next 6 days), and pain (during next 2 days).	None.
Yalcin 2008; Turkey (16)	DTwP (booster). N = 270. Age = 15–20 m.	Acetaminophen (10 mg/kg) along with vaccine (group 1), 2 hours after vaccination (group 2), and after the appearance of febrile reactions or irritability (group 3, control). In groups 1 and 2 in addition, if the axillary temperature was >38.0°C or if they were irritable, acetaminophen (10 mg/kg) was given, every 4 to 6 hr interval.	Local reaction (pain, redness and induration at the injection site), fever (≥38.0°C), high fever (≥39.0°C), and systemic reactions (drowsiness, loss of appetite, vomiting, diarrhea, and any other adverse events)	None.
Prymula 2009; Czech Republic (7)	Ten-valent pneumococcal non-typeable *H. influenzae* protein D-conjugate vaccine (PHiD-CV) co-administered with the hexavalent diphtheria-tetanus-3-component acellular pertussis-hepatitis B-inactivated poliovirus types 1, 2, and 3- *H. influenzae* type b (DTPa-HBV/IPV/Hib) and oral human rotavirus vaccines (both primary & booster). N = 459. Age = 9–16 wks, 12–15 m.	Three doses of paracetamol given within the first 24 h after each vaccine dose (first dose immediately after vaccination, second and third administrations were done at home every 6–8 hr). The dose was based on bodyweight: 80 mg/dose (53.3–34.3 mg/kg/24 h) for infants >4.5 kg and <7 kg, and 125 mg/dose (≤53.6 mg/kg/24 h) for infants ≥7 kg. At booster vaccination, the same dose was given to infants >7 kg and <9 kg, and those ≥9 kg received four administrations of 125 mg/dose (≤55.6 mg/kg/24 h).	Local symptoms (pain, redness, and swelling at the injection site), general symptoms (fever ≥38.0°C and >39.5°C, irritability/fussiness, drowsiness, and loss of appetite), vomiting and diarrhea. Immunogenicity was studied by measuring the antibody geometric mean concentrations (GMCs) of all vaccine types.	Antibody concentrations ≥0.20 µg/mL against pneumococcus serotype 6B; P vs C: 62.1% vs 75.6% (p<0.05). Antipneumococcal antibody GMCs against all ten vaccine serotypes: significantly lower in P group (p<0.05). Percentage of children with opsonophagocytic activity titres ≥8 for serotypes 1, 5, and 6B; P vs C: 34.8% vs 55.1% (p<0.05), 79.9% vs 93% (p<0.05), 82.2% vs 93.2% (p<0.05). Antiprotein D antibody GMC; P vs C: 985.4 U/mL vs 1599.1 ELISA U/mL (p<0.05). Seroprotection rates against *H. influenzae* type b at the 0.15 µg/mL, and 1.0 µg/mL cut-offs; P vs C: 96.1% vs 100% (p<0.05), and 73.9% vs 91.5% (p<0.05). GMCs for antibodies against *H. influenzae* type b, diphtheria, tetanus, and pertactin: significantly lower in P group (p<0.05). The effect of prophylactic paracetamol persisted after boosting similarly as above.
Prymula 2013; Czech Republic (8)	Ten-valent pneumococcal non-typeable *H. influenzae* protein D-conjugate vaccine (PHiD-CV) (booster). N = 220. Age = 31–44 m.	Follow up study to Prymula 2009 (7). No paracetamol used in the present study.	Antibody persistence, immunological memory and nasopharyngeal carriage (NPC) evaluated in this follow up study.	Induction of immunological memory was shown irrespective of prophylactic paracetamol (PP) administration. Antibody GMCs were lower in the PP group for serotypes 1, 4, 7F and 9V. Opsonophagocytic titres did not differ significantly between the two groups. No difference in the rate of NPC of vaccine pneumococcal serotypes and non-vaccine and non-cross-reactive serotypes were seen.
Prymula 2011; Czech Republic (17)	PHiD-CV (booster). N = 748. Age = 24–27 m.	Follow up study to Prymula 2009 (7). No paracetamol used in the present study.	Nasopharyngeal carriage (NPC) evaluated in this follow up study.	Carriage prevalence of pneumococcal vaccine serotypes; P vs C: 7.4% vs 6.8%, which was non-significant.
Jackson 2011; USA (18)	DTaP, DTaP-HepB-IPV, DTaP-IPV/Hib, HepB, Hib, Hib-HepB, IPV, PCV7, TIV (primary). N = 352. Age = 6 wks–10 m.	Acetaminophen 10–15 mg/kg/dose. First dose was given within an hr of vaccination or within the allowable window of 4 hrs before through up to 24 hrs after the vaccinations. A maximum of five doses should be given.	Primary outcome: Fever ≥38.0°C within 32 hrs following vaccinations. Secondary outcomes: medical utilization, fussiness, parents' time lost from work, and treatment assignment unblinded if child's symptom warrants supplementary acetaminophen treatment.	Fussiness; P vs C: 10% vs 24% (p<0.05). Unblinding of treatment assignment; P vs C: 3% vs 9% (p<0.05). Fever ≥38.0°C in infants ≥24 wks age; P vs C: 13% vs 25% (p = 0.03).
Hayat 2011; India (19)	DTwP, (both primary and booster). N = 302. Age = 6–14 wks, 18 m.	Acetaminophen 10 mg/kg/dose. First dose 1 hour before and then given at 6, 12 and 18 hours after vaccination.	Fever (≥38.0°C), local redness, local swelling/induration, local pain, refusal to feed, fussiness,	Fever ≥38.0°C; P vs C: 18.7% vs 55.3% (p<0.05). Fussiness; P vs C: 41.3% vs 74% (p<0.05). Unblinding of treatment assignment; P vs C: 3.3% vs 16.6% (p<0.01).
Rose 2013; Germany (20)	PCV-7 co-administered with hexavalent vaccine (DTPa-HBV-IPV/Hib) (both primary and booster). N = 301. Age = 56–112 days, 335–445 days	Paracetamol (125 mg or 250 mg suppositories, based on body weight) at vaccination, and at 6–8 hour intervals thereafter. Children <7 kg received 375 mg/day; children 7 to <10 kg received 500 mg/day; and children ≥10 kg received 750 mg/day.	Fever (≥38.0°C, >39.0°C, >40.0°C) tenderness, redness, swelling, rash irritability, drowsiness, decreased appetite, persistent inconsolable crying, decreased activity.	Fever ≥38.0°C (primary); P vs C: 43% vs 75.4% (p<0.05). Fever >39.0°C (booster); P vs C: 2.6% vs 12.2% (p<0.05). Rash (second dose, primary); P vs C: 6.8% vs15.7% (p = 0.04). Irritability (second and third dose, primary); P vs C: 47.2% vs 62.1% (p = 0.019) and 42.2% vs 58.5% 9 (p = 0.013). Drowsiness (first dose, primary); P vs C: 50.4% vs 64.7% (p = 0.019). Decreased appetite (second dose, primary); P vs C: 26.6% vs 42.7% (p = 0.011). Persistent inconsolable crying (first dose, primary); P vs C: 9.5% vs 20% (p = 0.031). Persistent inconsolable crying (booster); P vs C: 7.8% vs 17.1% (p = 0.05). Decreased activity (second and third dose, primary); P vs C: 31% vs 48% (p = 0.007) and 23.3% vs 40% (p = 0.007). Decreased activity (booster); P vs C: 29% vs 48.3% (p = 0.005).
Wysocki 2014; USA (21)	PCV13 co-administered with DTaP/IPV/Hib/HBV (primary). N = 908. Age = 2–4 and 12 months.	Paracetamol (15 mg/kg/dose) at vaccination, at 6–8 hr, and 12–16 hr. Ibuprofen (10 mg/kg/dose) at vaccination, at 6–8 hr, and 12–16 hr. Five groups (2 groups received paracatemol or ibuprofen at vaccination and thereafter, 2 groups did not receive paracatemol or ibuprofen at vaccination but thereafter, one control group did not receive any of these).	Antibody/immune response to all the administered vaccine antigens.	Pneumococcal anticapsular IgG geometric GMCs were significantly (p<0.0125) lower in G3 (received paracetamol at vaccination) versus G5 (control) for 5 of 13 serotypes after the primary series. Pertussis FHA and tetanus IgG GMC were significantly lower among G4 (received ibuprofen at vaccination) versus G5 (control) after the primary series. No differences were observed for any antigens after the toddler dose.

### Data synthesis including assessment of heterogeneity

The data from various studies were pooled and expressed as, odds ratio (OR) with 95% confidence interval (CI) for dichotomous data, and mean difference (MD) with 95% CI for continuous data. A p-value of <0.05 was considered significant. Assessment of heterogeneity was done by I-squared (I^2^) statistics. If there was a high level of heterogeneity (>50%), we tried to explore this by subgroup analysis if there were adequate number of trials. A fixed effects model was initially conducted. If significant heterogeneity existed among trials (>50%), potential sources of heterogeneity were considered, and where appropriate, a random effects model was used. *RevMan (Review Manager) version 5.2* was used for all the analyses [22].

## Results

### Description of studies

**Figure 1:**
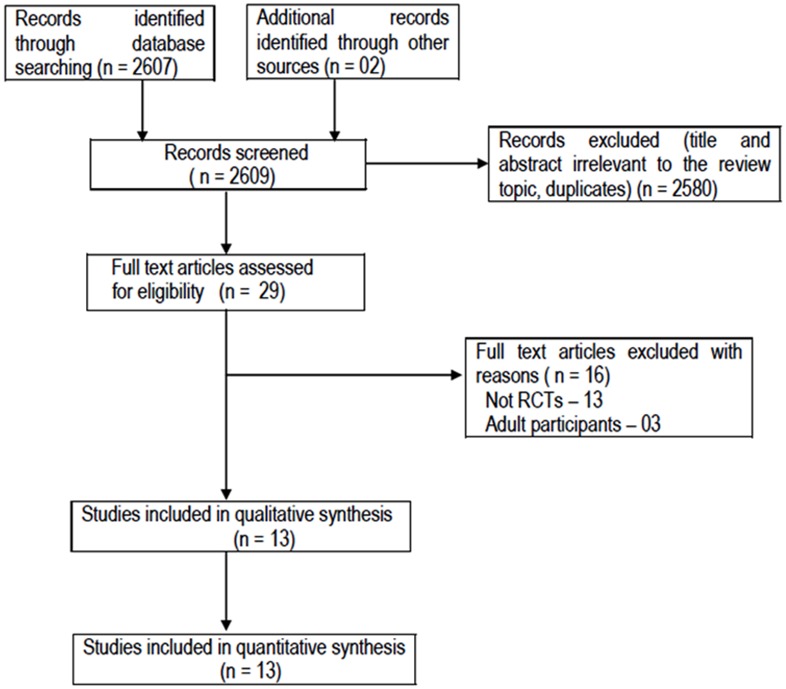
Study flow.

Of 2579 citations retrieved, full text of 26 articles were assessed for eligibility (**Figure 1**). Out of these, a total of 14 articles were excluded for the following reasons: non RCTs (n = 11), adult participants (n = 03). Finally, 13 trials including 5077 children were included in the review (**Table 1**) [7], [8], [11]–[21]. The included trials were conducted in both developed (USA = 4, Czech Republic  = 3, Canada  = 1, Germany  = 1, Turkey  = 1, Finland  = 1, and Spain  = 1) and developing countries (India  = 1). One trial used ibuprofen [14], two used both paracetamol and ibuprofen [15],[21], and others used only paracetamol [7], [8], [11]–[13], [16]–[20]. The trials were heterogeneous regarding the dosage schedule of intervention, the age of the enrolled children, type of vaccine used, and the outcomes measured. Children >1 months (not neonates) were included in the studies. Isolated DTwP vaccine was used in six trials [11]–[14], [16], [19], isolated DTaP in one trial [15], and rest others used combination vaccine [7], [8], [17], [18], [20], [21].

### Risk of bias in included studies

All the included trials had moderate to high risk of bias because of the following reasons: open or single-blind nature, small sample size, and other sources of bias.

### Effect of prophylactic Paracetamol (PCM)

#### Primary outcome measures

**Figure 2:**
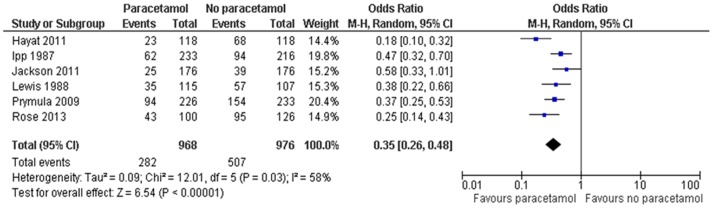
Prophylactic paracetamol: febrile reactions ≥38.0°C (100.4°F) in the first 24–48 hrs after primary vaccination.

**Figure 3:**
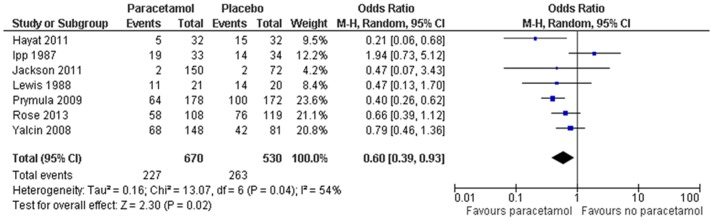
Prophylactic paracetamol: febrile reactions ≥38.0°C (100.4°F) in the first 24–48 hrs after booster vaccination.

(1) Febrile reactions ≥38.0°C (100.4°F) in the first 24–48 hrs: Compared to the no prophylactic PCM group, there was a significant reduction in the febrile reactions of ≥38.0°C (100.4°F) in the first 24–48 hrs in the prophylactic PCM group, both after primary [OR, 0.35; 95%CI, 0.26–0.48] (**Figure 2**) and booster [OR, 0.60; 95%CI, 0.39–0.93] (**Figure 3**) vaccinations. However, because of a high degree of heterogeneity (>50%), these results should be interpreted with caution.

(2) Antibody response rate (measured by GMCs) after primary vaccination (3, 4, and 5 months age): There was significant difference in the GMC of the anti-pneumococcal IgG antibody between the prophylactic PCM group and no prophylactic PCM group, for all the vaccine serotypes: serotype 1 [MD −0.53 (95%CI, −0.71 to −0.35)], serotype 4 [MD −0.8 (95%CI, −1.08 to −0.52)], serotype 5 [MD −0.62 (95%CI, −0.87 to −0.37)], serotype 6B [MD −0.2 (95%CI, −0.29 to −0.11)], serotype 7F [MD −0.59 (95%CI, −0.83 to −0.35)], serotype 9V [MD −0.46 (95%CI, −0.65 to −0.27)], serotype 14 [MD −1.28 (95%CI, −1.79 to −0.77)], serotype 18C [MD −1.47 (95%CI, −1.82 to −1.12)], serotype 19F [MD −2.13 (95%CI, −2.93 to −1.33)], and serotype 23F [MD −0.27 (95%CI, −0.43 to −0.11)]. Regarding other vaccinations, there was significant difference in the GMC of the anti-PRP [MD −1.99 (95%CI, −2.76 to −1.22)], anti-diphtheria [MD −0.89 (95%CI, −1.27 to −0.51)], anti-tetanus [MD −1.04 (95%CI, −1.34 to −0.74)], anti-pertactin [MD −27.9 (95%CI, −38.65 to −17.15)] between the prophylactic PCM group and no prophylactic PCM group. The GMC of anti-PT, anti-FHA, anti-HBs, and anti-polio (type 1,2,3) did not show any significant difference between the prophylactic PCM group and no prophylactic PCM group. Though the GMC of all pneumococcal vaccines serotypes and some other vaccines decreased after prophylactic PCM, still the level of GMC in the prophylactic PCM group was well above the seroprotection level.

(3) Antibody response rate (measured by GMCs) after first booster vaccination (12–15 months age): There was significant difference in the GMC of the anti-pneumococcal IgG antibody between the prophylactic PCM group and no prophylactic PCM group, for all the vaccine serotypes: serotype 1 [MD −0.96 (95%CI, −1.37 to −0.55)], serotype 4 [MD −1.22 (95%CI, −1.84 to −0.6)], serotype 5 [MD −1.38 (95%CI, −1.91 to −0.85)], serotype 6B [MD −1.11 (95%CI, −1.5 to −0.72)], serotype 7F [MD −1.24 (95%CI, −1.81 to −0.67)], serotype 9V [MD −1.55 (95%CI, −2.17 to −0.93)], serotype 14 [MD −1.38 (95%CI, −2.28 to −0.48)], serotype 18C [MD −2.03 (95%CI, −2.99 to −1.07)], serotype 19F [MD −1.55 (95%CI, −3.08 to −0.02)], and serotype 23F [MD −0.93 (95%CI, −1.5 to −0.36)]. Regarding other vaccinations, there was significant difference in the GMC of the anti-diphtheria [MD −2.2 (95%CI, −3.82 to −0.58)], anti-tetanus [MD −2.2 (95%CI, −3.25 to −1.15)] between the prophylactic PCM group and no prophylactic PCM group. The GMC of anti-PRP, anti-PT, anti-FHA, anti-pertactin, anti-HBs, and anti-polio (type 1,2,3) did not show any significant difference between the prophylactic PCM group and no prophylactic PCM group. Though the GMC of all pneumococcal vaccines serotypes and some other vaccines decreased after prophylactic PCM, still the level of GMC in the prophylactic PCM group was well above the seroprotection level.

(4) Antibody response rate (measured by GMCs) after second booster vaccination (40–48 months age): There was significant difference in the GMC of the anti-pneumococcal IgG antibody between the prophylactic PCM and no prophylactic PCM group, for the following vaccine serotypes: serotype 1 [MD −4.27 (95%CI, −6.75 to −1.79)], serotype 4 [MD −4.78 (95%CI, −8.16 to −1.4)], serotype 5 [MD −3.69 (95%CI, −6.67 to −0.71)], serotype 7F [MD −2.92 (95%CI, −4.74 to −1.1)], serotype 9V [MD −4.59 (95%CI, −7.4 to −1.78)], serotype 14 [MD −6.7 (95%CI, −13.35 to −0.05)], serotype 18C [MD −12.54 (95%CI, −22.1 to −2.98)]. The GMC of the anti-pneumococcal IgG antibody for serotypes 6B, 19F, and 23F did not show statistically significant difference between the prophylactic PCM and no prophylactic PCM group. No study reported this outcome for other vaccinations.

#### Secondary outcome measures

(1) High febrile reactions ≥39.0°C in the first 24–48 hrs: compared to the placebo group, there was a significant reduction in the high febrile reactions of ≥39.0°C in the first 24–48 hrs in the prophylactic PCM group after primary [OR, 0.31; 95%CI, 0.18–0.52], but not booster [OR, 0.63; 95%CI, 0.35–1.11] vaccinations.

(2) Pain of all grades: compared to the no prophylactic PCM group, there was a significant reduction in the pain of all grades in the prophylactic PCM group, both after primary [OR, 0.57; 95%CI, 0.47–0.7] and booster [OR, 0.64; 95%CI, 0.48–0.84] vaccinations.

(3) Pain of moderate to severe grade: compared to the no prophylactic PCM group, there was a significant reduction in the pain of moderate to severe grade in the prophylactic PCM group after primary [OR, 0.39; 95%CI, 0.26–0.58], but not booster [OR, 0.59; 95%CI, 0.24–1.45] vaccinations.

(4) Local redness: compared to the no prophylactic PCM group, there was a significant reduction in the local redness in the prophylactic PCM group after primary [OR, 0.81; 95%CI, 0.68–0.95], but not booster [OR, 0.93; 95%CI, 0.73–1.18] vaccinations.

(5) Local swelling/induration: compared to the no prophylactic PCM group, there was a significant reduction in the local swelling/induration in the prophylactic PCM group after primary [OR, 0.78; 95%CI, 0.66–0.92], but not booster [OR, 0.90; 95%CI, 0.68–1.19] vaccinations.

(6) Persistent cry: compared to the no prophylactic PCM group, there was a significant reduction in the rate of persistent cry in the prophylactic PCM group, both after primary [OR, 0.55; 95%CI, 0.39–0.77] and booster [OR, 0.44; 95%CI, 0.22–0.87] vaccinations.

(7) Irritability/fussiness: compared to the no prophylactic PCM group, there was a significant reduction in the irritability/fussiness in the prophylactic PCM group, both after primary [OR, 0.36; 95%CI, 0.29–0.45] and booster [OR, 0.66; 95%CI, 0.48–0.91] vaccinations.

(8) Drowsiness: compared to the no prophylactic PCM group, there was a significant reduction in the drowsiness in the prophylactic PCM group after primary [OR, 0.82; 95%CI, 0.70–0.96], but not booster [OR, 0.99; 95%CI, 0.76–1.3] vaccinations.

(9) Anorexia/loss of appetite: compared to the no prophylactic PCM group, there was a significant reduction in the anorexia/loss of appetite in the prophylactic PCM group after primary [OR, 0.61; 95%CI, 0.49–0.77], but not booster [OR, 0.85; 95%CI, 0.64–1.14] vaccinations.

(10) Vomiting: There was no significant difference between the prophylactic PCM and the no prophylactic PCM group regarding the reduction of vomiting.

(11) Diarrhea: There was no significant difference between the prophylactic PCM and the no prophylactic PCM group regarding the reduction of diarrhea.

(12) Any severe symptom: compared to the no prophylactic PCM group, there was a significant reduction in any severe symptom in the prophylactic PCM group after booster [OR, 0.38; 95%CI, 0.20–0.71], but not primary [OR, 0.81; 95%CI, 0.58–1.12] vaccinations.

(13) Nasopharyngeal carriage (NPC) rate of the organisms (*S. pneumoniae*, *H. influenzae*, and others)

There was no significant difference either in the pneumococcal carriage rate (any serotype, vaccine serotypes, or any cross-reactive serotype) or in the H. influenza carriage rate between the prophylactic PCM and the no prophylactic PCM group. The significant finding of post-booster non-typeable *H. influenzae* carriage rate [OR, 0.61; 95%CI, 0.39–0.95] might be due to chance or inadequate randomization.

(14) Days of parental work loss

There was no significant difference between the prophylactic PCM and no prophylactic PCM group for the days of parental work loss.

### Effect of prophylactic Ibuprofen (IB)

#### Primary outcome measures

(1) Febrile reactions ≥38.0°C (100.4°F) in the first 24–48 hrs: there was no significant difference between the prophylactic IB and no prophylactic IB groups regarding the reduction of febrile reactions ≥38.0°C (100.4°F) in the first 24–48 hrs of primary and booster vaccinations.

#### Secondary outcome measures

(1) High febrile reactions ≥39.0°C in the first 24–48 hrs: there was no significant difference between the prophylactic IB and no prophylactic IB group regarding the reduction of febrile reactions ≥39.0°C in the first 24–48 hrs of primary vaccination.

(2) Pain all grades: compared to the prophylactic IB group, there was a significant increase in the pain of all grades in the no prophylactic IB group after primary [OR, 1.52; 95%CI, 1.13–2.04], but not booster [OR, 0.97; 95%CI, 0.55–1.7] vaccinations.

(3) Pain (moderate to severe): compared to the prophylactic IB group, there was a significant increase in the moderate to severe pain in the no prophylactic IB group after primary [OR, 1.73; 95%CI, 1.1–2.72], but not booster [OR, 0.95; 95%CI, 0.41–2.24] vaccinations.

(4) Local redness: There was no significant difference between the prophylactic IB and no prophylactic IB group regarding the reduction of local redness after primary and booster vaccinations.

(5) Swelling/induration: compared to the prophylactic IB group, there was a significant increase in the swelling/induration in the no prophylactic IB group after primary [OR, 1.44; 95%CI, 1.06–1.94] vaccination.

(6) Prolonged cry: There was no significant difference between the prophylactic IB and no prophylactic IB group regarding prolonged cry after primary vaccination.

(7) Irritability/fussiness: There was no significant difference between the prophylactic IB and no prophylactic IB group regarding irritability/fussiness after primary vaccination.

(8) Drowsiness: compared to the prophylactic IB group, there was a significant increase in drowsiness in the no prophylactic IB group after primary [OR, 1.36; 95%CI, 1.00–1.86] vaccination.

(9) Anorexia/loss of appetite: There was no significant difference between the prophylactic IB and no prophylactic IB group regarding anorexia/loss of appetite after primary vaccination.

(10) Vomiting: There was no significant difference between the prophylactic IB and no prophylactic IB group regarding vomiting after primary vaccination.

(11) Diarrhea: There was no significant difference between the prophylactic IB and no prophylactic IB group regarding diarrhea after primary vaccination.

### Effect of prophylactic PCM and prophylactic IB

#### Primary outcome measure

(1) Antibody response rate (measured by GMCs) after primary vaccination (2, 3, 4, and 12 month age): This was reported in one trial [presented as conference abstract]. The trial employed 5 groups (**Table 1**), and the results were as follows. Pneumococcal anticapsular IgG GMCs were significantly lower (p<0.0125) in G3 (received paracetamol at vaccination and thereafter) versus G5 (no antipyretic) for 5 of 13 serotypes after the primary series. Pertussis FHA and tetanus IgG GMC was significantly lower among G4 (received ibuprofen at vaccination and thereafter) versus G5 (no antipyretic) after the primary series. No differences were observed for any antigens after the toddler dose. The trial concluded that prophylactic PCM may interfere with primary series immune response to pneumococcal antigens. Prophylactic IB did not interfere with pneumococcal responses, but may reduce response to pertussis FHA and tetanus antigens. These effects were not observed following the toddler dose. The clinical significance of these findings is unclear.

### Publication bias

**Figure 4:**
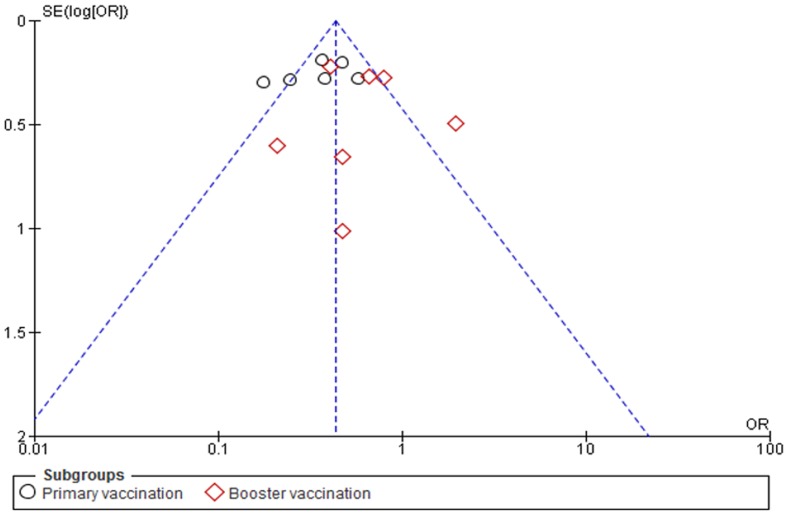
Funnel plot for assessing publication bias by including studies reporting the primary outcome.

To assess whether there was a bias in the published literature, funnel plot was constructed using the OR and 1/SE values obtained from studies measuring the primary outcome (febrile reactions of ≥38.0°C in the first 24–48 hrs of PCM administration). In the absence of a publication bias, such a plot is expected to have a shape resembling an inverted funnel [23]. From the asymmetry of funnel plot generated, the possibility of publication bias in the analysis cannot be ruled out (**Figure 4**).

## Discussion

### Summary of evidence

Prophylactic antipyretic administration significantly reduced the febrile reactions of ≥38.0°C after primary and booster vaccinations. Though there were statistically significant differences in the antibody responses between the two groups (being lower in the prophylactic PCM group), the prophylactic PCM group had what would be considered protective levels of antibodies (GMCs) to all of the antigens given after the primary and booster vaccinations. There was a significant reduction in the local and systemic symptoms after primary, but not booster vaccinations (except for any severe symptom, that had a significant reduction after booster but not primary vaccinations).

The present review does not find a strong evidence to support the conclusion of a well conducted RCT that questioned the administration of prophylactic PCM during administration of childhood vaccines. This RCT had concluded that although febrile reactions significantly decreased, prophylactic administration of PCM at the time of vaccination should not be routinely recommended since antibody responses to several vaccine antigens were reduced [7]. However, since the antibody response (GMC) was not reduced below seroprotection level, it is unlikely that prophylactic PCM would have any detrimental effect for individual child concerned. The same has been endorsed by AAP in their guidelines [9]. Regarding the new trial studying the effect of PCM and IB simultaneously, the results are more complicated, as it found differential effect of the antipyretics on the vaccine antigen responses [21].

The present review finds a benefit in favour of prophylactic antipyretic administration on both local and systemic symptoms post-vaccination, although the analyses included trials using mostly DTwP (6 trials) instead of DTaP (3 trials), the later being less reactive. The results of the RCT that has sparked the debate about the beneficial role of prophylactic antipyretic though cannot be ignored, but cannot be accepted with foolproof at the same time [7]. This is because of the following four points. First, there is only a small decrease in the GMC of vaccine antibody titers that may be of statistically significant but the clinical/epidemiological relevance is not clear. The latter is supported by the fact that, in spite of being a common practice for administration of prophylactic antipyretics after immunizations for decades, there have been significant reductions in invasive disease due to *S. pneumoniae* and *H. influenzae* type b serotypes. Second, the follow up study to the above RCT has shown that regardless of the administration of prophylactic PCM, there was no effect on the nasopharyngeal carriage rate post-booster vaccination [8]. Third, the development of fever or increase in the temperature post-vaccination due to the release of endogenous cytokines (IL 1, TNF α), has been considered as a marker of immune response to respected vaccines. Fourth, the potential interference between different vaccines when co-administered with or without antipyretics should also be taken into consideration. For example, 30–60% lower anti-HBs GMTs occur when co-administered with HPV vaccines and that without antipyretics, which might further diminish the magnitude of the immune response. It has also been seen that the acellular pertussis vaccine is much less immunogenic than the whole cell, and PCV13 develops lower IgG concentrations than PCV7 to the common serotypes. If this is already the case, adding prophylactic PCM that could lower the immune response even lower, could be a problem. If there is already herd immunity, maybe a small decrease in efficacy at the individual level will take a long time to be noticed, and would raise the need for better surveillance programs for vaccine-preventable diseases in all countries. Because of these later two findings, there is concern that prophylactic antipyretic might decrease the post-vaccination immune response further.

Besides these, the findings of another RCT [24] reporting about the infant sleep after immunization and relation of acetaminophen (paracetamol) use need mention here. This RCT found that paracetamol use post-immunization (not prophylactic) was associated with increase in the infant sleep duration. As sleep deprivation before or after has been associated with decreased antibody formation post-immunization in adults, this study postulates that use of acetaminophen post-immunization might facilitate the immune response. But this study neither studied the effect of prophylactic antipyretic nor measured the antibody response.

### Limitations

Only two trials (from the same country) studied the antibody response (one trial) and carriage rate (one trial) as a result the data could not be pooled. Studies used different doses/schedules of antipyretic administration resulting in significant heterogeneity in the pooled result. The age of the participants or timing of administration also markedly differed among the studies. Only one study from developing country (India) made it difficulty in generalizing the present review findings.

### Further area of research

Future trials should focus on the timing (before, with or after) and route (oral or rectal) of administration of paracetamol as well as on the subgroup of infants (term or preterm) for any correlation with the immune response. As there was no trial examining the prophylactic effect of ibuprofen on post-vaccination antibody response, future trials should focus on this. Any post-vaccination decrease in antibody titer noted in future studies should be correlated with the natural history of that particular disease. The mechanism underlying the decrease in immune/antibody response should also be explored. Immune response to varicella, hepatitis A, measles, MMR, and flu vaccine should also be studied, if feasible. Trials should also be conducted in developing countries where over-the-counter use of antipyretics (including prophylactic) are common. Other confounding factors that might affect the antibody response (e.g., infant sleep post-immunization) should also be studied.

## Conclusions

Though prophylactic antipyretic administration leads to relief of the local and systemic symptoms after primary vaccinations, there is a reduction in antibody responses to some vaccine antigens without any effect on the nasopharyngeal carriage rates of *S. pneumoniae* & *H. influenza* serotypes. Future trials and surveillance programs should also aim at assessing the effectiveness of programs where prophylactic administration of PCM is given. The timing of administration of antipyretics should be discussed with the parents after explaining the benefits & risks.

## Supporting Information

Checklist S1 **PRISMA checklist.**
(DOC)

Appendix S1 **Detailed search strategy.**
(DOC)
