# Supplementary material for: The Effect of Prophylactic Antipyretic Administration on Post-Vaccination Adverse Reactions and Antibody Response in Children: A Systematic Review
Source: PLoS One. 2014 Sep 2;9(9):e106629. doi: 10.1371/journal.pone.0106629 (PMC4152293; doi:10.1371/journal.pone.0106629)
Supplement: Appendix S1 — Detailed search strategy. (DOC) [file pone.0106629.s002.doc]

**Appendix I**

**PUBMED**

1. ("antipyretics"[MeSH Terms] OR ("antipyretic"[All Fields] AND "prophylactic"[All Fields]) OR "prophylactic antipyretic"[All Fields] OR "paracetamol"[All Fields]) AND ibuprofen[All Fields] AND ("vaccination"[MeSH Terms] OR "immunization"[All Fields])
2. ("prophylactic antipyretic "[MeSH Terms] OR ("prophylactic"[All Fields] AND " prophylactic antipyretic "[All Fields]) OR " prophylactic antipyretic "[All Fields] OR "healthcare"[All Fields]) AND ("adverse event"[MeSH Terms] OR "adverse events"[All Fields])
3. "post-vaccination"[MeSH Terms] OR "post-immunization"[All Fields]
4. "local"[MeSH Terms] OR "localized"[All Fields]
5. "generalized"[MeSH Terms] OR "systemic"[All Fields]
6. (#2) AND #3
7. (#1) AND #4
8. (#1) AND #5
9. "child"[MeSH Terms] OR "child"[All Fields] OR "children"[All Fields]
10. "infant"[MeSH Terms] OR "infants"[All Fields]
11. (#6) AND #7
12. ((#9) AND #10
13. (#11) OR ("streptococcus pneumoniae"[MeSH Terms] OR ("pneumococcus"[All Fields])
14. (#12) AND ("streptococcus pneumoniae"[MeSH Terms] OR ("pneumococcus"[All Fields])
15. (#11) AND ("streptococcus pneumoniae"[MeSH Terms] OR ("pneumococcus"[All Fields])
16. (#12) OR ("hemophilus influenzae"[MeSH Terms] OR ("hemophilus"[All Fields] AND " influenzae "[All Fields])
17. (#11) AND ("hemophilus influenzae"[MeSH Terms] OR ("hemophilus"[All Fields] AND "influenzae "[All Fields])
18. (#11) OR ("hemophilus influenzae"[MeSH Terms] OR ("hemophilus"[All Fields] AND "influenzae"[All Fields])
19. (#12) AND ("hemophilus influenzae"[MeSH Terms] OR ("hemophilus"[All Fields] AND "influenzae"[All Fields])
20. (#11) AND ("DPwT"[MeSH Terms] OR "DPT"[All Fields] OR “whole cell vaccine” [All Fields])
21. (#12) OR ("DPwT"[MeSH Terms] OR "DPT"[All Fields] OR “whole cell vaccine” [All Fields])
22. (#11) OR ("DTaP"[MeSH Terms] OR "DTap"[All Fields]) OR “acellular vaccine” [All Fields]
23. (#12) AND ("DTaP"[MeSH Terms] OR "DTap"[All Fields]) OR “acellular vaccine” [All Fields]
24. (#13-23) AND #11
25. (#13-23) AND #12
26. (#24) AND "Conjugate vaccine"[All Fields]
27. (#25) AND "Conjugate vaccine"[All Fields]
28. (#24) AND "PCV-7"[All Fields]
29. (#25) AND "PCV-7"[All Fields]
30. (#24) AND "PCV-10"[All Fields]
31. (#25) AND "PCV-10"[All Fields
32. (#24) AND "PHiD-CV"[All Fields]
33. (#25) AND "PHiD-CV"[All Fields]
34. (#24) AND "DTPa-HBV-IPV/Hib"[All Fields]
35. (#25) AND "DTPa-HBV-IPV/Hib"[All Fields]
36. (#24) AND "MMR"[All Fields]
37. (#25) AND "MMR"[All Fields]
38. (#24) AND "Rotavirus"[All Fields]
39. (#25) AND "Rotavirus"[All Fields]
40. (#24) AND "IPV"[All Fields]
41. (#25) AND "IPV"[All Fields]
42. (#24) AND "Meningococcal conjugate"[All Fields]
43. (#25) AND "Meningococcal conjugate"[All Fields]
44. (#24) AND "Hepatitis vaccine"[All Fields]
45. (#25) AND "Hepatitis vaccine"[All Fields]
46. (#24) AND "Varicella vaccine"[All Fields]
47. (#25) AND "Varicella vaccine"[All Fields]
48. (#24) AND "Polysaccharide vaccine"[All Fields]
49. (#25) AND "Polysaccharide vaccine"[All Fields]

**EMBASE**

32. #8 AND #31

31. 'rota virus'/exp OR 'rota virus vaccine'

30. #8 AND #29

29. 'injectable polio'/exp OR 'injectable polio vaccine'

28. #8 AND #27

27. 'polysaccharide vaccine'/exp OR 'polysaccharide vaccine'

26. #8 AND #25

25. 'meningococcal vaccine'/exp OR 'meningococcal conjugate vaccine'

24. #8 AND #23

23. 'varicella'/exp OR 'varicella vaccine'

22. #8 AND #21

21. 'hepatitis'/exp OR 'hepatitis vaccine'

20. #8 AND #19

19. 'MMR'/exp OR 'mumps-measles-rubella'

18. #8 AND #17

17. 'DTPa-HBV-IPV/Hib'/exp OR 'hexavalent diphtheria-tetanus-3-component acellular pertussis-hepatitis B-inactivated poliovirus types 1, 2, and 3-H. influenzae type b'

16. #8 AND #15

15. 'PHiD-CV-13'/exp OR '10-valent pneumococcal non-typeable H. influenzae protein D-conjugate vaccines'

14. #8 AND #13

13. 'PCV-10'/exp OR 'pneumococcal 10-valent conjugate'

12. #8 AND #11

11. 'PCV-7'/exp OR 'pneumococcal 7-valent conjugate'

10. #8 AND #9

9. 'health care'/exp OR 'health care facility'

8. #4 OR #6

7. #2 AND #6

6. 'post-vaccination' AND ('immunize'/exp OR immunization)

5. 'post-vaccination'

4. #2 AND #3

3. 'analgesic'/exp OR 'analgesic-antipyretic'

2. 'child'/exp OR child AND ('children'/exp OR children) OR 'infant'/exp OR infant OR pediatric

1. 'antipyretic'/exp OR antipyretic
